# Assessment of emicizumab levels in EDTA plasma

**DOI:** 10.1016/j.rpth.2025.103175

**Published:** 2025-09-05

**Authors:** Christian Pfrepper, Annelie Siegemund, Tristan Klöter, Hagen Bönigk, Sirak Petros, Thomas Siegemund

**Affiliations:** 1 Division of Hemostaseology, University of Leipzig Medical Center, Leipzig, Germany; 2 MVZ Limbach Magdeburg, Magdeburg, Germany; 3 Medical ICU, University Hospital Leipzig, Leipzig, Germany

To the Editor,

Emicizumab is a bispecific antibody that mimics the role of activated coagulation factor (F)VIII by combining activated FIX with FX and leading to the activation of FX [1]. Emicizumab is approved for bleeding prophylaxis in patients with severe hemophilia A (HA) with and without inhibitors and in patients with moderate HA with severe bleeding type [[2], [3], [4]].

Emicizumab plasma levels can be measured using mass spectrometry a time-consuming and expensive technology rarely accessible outside specialized laboratories. Therefore, a modified functional FVIII assay (mOSA) calibrated against emicizumab is used as the standard assay to determine emicizumab plasma levels [5]. Although the mOSA quantifies the FVIII-like activity of emicizumab with a higher predilution than a conventional FVIII assay, residual FVIII in the plasma sample leads to falsely elevated emicizumab concentrations [6]. Heat inactivation of FVIII was discussed to overcome this limitation, but emicizumab concentration was lowered by 40% to 50% during this procedure when mOSA was used [7]. Another approach, inactivation of FVIII by neutralizing inhibitors, may lead to acceptable results in spiked plasma samples with mOSA. However, inactivation of FVIII can only be performed in specialized laboratories, making this method unlikely to be used in routine clinical practice. In addition, emicizumab plasma levels can be determined by 2-stage chromogenic assays, providing reliable results even after heat inactivation of FVIII [8]. However, this approach is rarely used in routine clinical testing. Therefore, an easy-to-perform method for the accurate measurement of emicizumab plasma levels in the presence of FVIII is highly warranted.

The aim of our study was to show that emicizumab can be measured in EDTA-containing plasma, while FVIII is not stable in such an environment. If this approach is valid, the measurement of emicizumab in EDTA plasma using mOSA leads to comparable results as in citrated plasma without relevant influence of FVIII. EDTA (and serum) is a standard sampling material used in clinical laboratories to quantify or test for antibodies.

Blood samples for all measurements were collected in citrate or K3-EDTA–containing tubes at a concentration of 4.1 mmol/L (Sarstedt). Samples were centrifuged at 2000*g* for 20 minutes and stored within 4 hours at −70 °C. Samples were thawed at 37 °C immediately before measurement. Factor VIII levels were assessed using the 1-stage clotting assay with Actin FS as activator (Siemens Healthineers). Emicizumab levels were determined by the modified functional FVIII assay using Actin FS as activator and a dedicated emicizumab calibrator (Haemochrom Diagnostica). The procedure of the test is given in Supplementary Methods. All assays were performed on an Atellica COAG 360 as well as a CN-6000 analyzer (both Siemens Healthineers).

Leftover samples from 20 patients presenting at the MVZ Limbach Magdeburg (Magdeburg, Germany) for hemostatic counseling were included to demonstrate the inactivation of FVIII in EDTA plasma. Emicizumab plasma levels were measured in patients with HA on long-term prophylaxis with emicizumab from the Hemophilia Center of the University Hospital Leipzig, Germany. Spearman correlation coefficient *r* and *P* values were determined for the correlation of emicizumab plasma levels in citrated and EDTA plasma. The coefficient of variation was determined for all subsequent laboratory measurements. In addition, Passing–Bablok analysis and Bland–Altman plots were performed to compare emicizumab plasma concentration in citrated plasma and EDTA on COAG 360 and CN-6000. Microsoft Excel and GraphPad Prism (version 10.4.2; GraphPad Software) were used for the statistical analysis and to generate graphs. All patients provided written informed consent, and the study was approved by the Ethics Committee of the University of Leipzig (ref. 355/24-ek) and conducted in accordance with the Declaration of Helsinki.

FVIII was determined simultaneously in citrated and EDTA plasma. The mean inhibition of the FVIII activity measured in EDTA plasma was 98.3% (SD, 2.4%; range, 93.5%-100%) on the COAG 360 platform and 94.5% (SD, 4.8%; range, 86.9%-100%) on CN-6000, respectively. The highest residual FVIII activity in EDTA was 10.5 IU/dL. All measurements are shown in the Table.

**Table:** Factor VIII activity (IU/dL) in citrated plasma and EDTA and degree of inhibition measured on COAG 360 and CN-6000.

Plasma sample	COAG 360			CN-6000		
	Citrated plasma	EDTA	Inhibition (%)	Citrated plasma	EDTA	Inhibition (%)
1	24.7	ND	100	25.0	0.5	98.0
2	49.4	ND	100	45.7	ND	100
3	54.6	1.8	96.7	42.9	5.6	86.9
4	61.9	ND	100	51.1	ND	100
5	75.2	ND	100	65.8	ND	100
6	78.7	ND	100	76.4	8.2	89.3
7	80.5	5.2	93.5	76.4	7.9	89.7
8	83.6	ND	100	81.3	ND	100
9	90.0	ND	100	92.2	6.2	93.3
10	91.1	1.0	98.9	83.9	ND	100
11	94.4	ND	100	91.3	ND	100
12	96.2	ND	100	88.0	7.3	91.7
13	96.7	4.9	94.9	93.7	9.2	90.2
14	106.9	ND	100	90.8	ND	100
15	110.7	ND	100	103.0	ND	100
16	111.6	ND	100	104.6	9.5	90.9
17	113.5	5.7	95.0	101.3	ND	90.1
18	121.3	5.1	95.8	104.6	8.7	91.7
19	122.0	3.9	96.8	115.9	7.2	93.8
20	129.9	7.0	94.6	120.5	10.5	91.3

ND, not detectable (<0.3 IU/dL).

Twenty-five plasma samples from 14 patients with congenital HA (3 severe HA with inhibitor, 8 severe HA without inhibitors, and 3 moderate HA without inhibitors) and 2 patients with acquired HA from the Hemophilia Center of the University Hospital Leipzig, Germany, on prophylaxis with emicizumab were included for the determination of emicizumab plasma levels in citrated plasma and EDTA.

The Spearman correlation between the emicizumab plasma concentration measured in citrated plasma and that in EDTA plasma was 0.997 for COAG 360 and 0.984 for CN-6000. The correlation between the emicizumab plasma levels measured on COAG 360 and CN-6000 was 0.968 in citrated plasma and 0.952 in EDTA. Passing–Bablok analysis showed a slope of 1.00 (range, 0.97-1.04) and intercept of −1.05 μg/mL (range, −3.29 to 0.88) on the COAG 360 platform and a slope of 1.06 (range, 0.97-1.13) and intercept of 0.19 μg/mL (range, −3.94 to 4.16) on the CN-6000 analyzer (Supplementary Figure 1).

The relative difference for the measurement of emicizumab plasma levels in EDTA vs citrated plasma ranged between −6.5% and +2.0% on COAG 360 and between −4.9% and +8.8% on CN-6000 (Supplementary Table; Supplementary Figure 2). The Figure shows the Bland–Altman plot of the emicizumab plasma concentration measured in citrated plasma and EDTA on COAG 360 and CN-6000.

**Figure:**
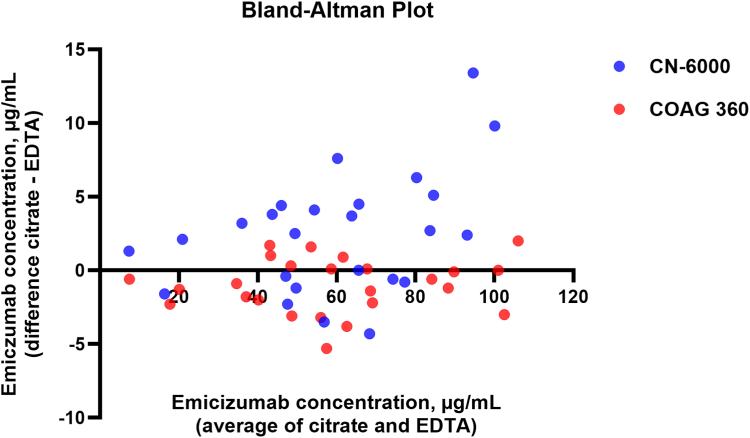
Bland–Altman Plot of the emicizumab plasma concentration measured in citrated plasma and EDTA on COAG 360 and CN-6000.

This study aimed to show that emicizumab plasma levels can be reliably measured in EDTA plasma without a relevant influence of FVIII. We were able to show that EDTA inhibits FVIII activity by more than 86% at clinically relevant FVIII levels between 25 and 130 IU/dL. EDTA irreversibly chelates calcium and leads to permanent blockade of the coagulation cascade. Decades ago, it has been shown that EDTA inactivates FVIII through the chelation of the intrinsic calcium of FVIII [9]. While FVIII antigen is still preserved in EDTA plasma, FVIII activity is markedly reduced [10]. Hamedani et al. [7] have shown that the emicizumab plasma levels increase by approximately 0.12 μg/mL per IU/dL of FVIII [7]. The highest residual FVIII activity after EDTA pretreatment was 10.5%, which would lead to an overestimation of approximately 1.2 μg/mL emicizumab plasma concentration. The spiking experiments performed by Bowyer et al. [6] showed a much higher increase of the emicizumab plasma concentration when emicizumab containing plasma at 25, 50, and 75 μg/mL was spiked with different recombinant FVIII concentrates at 50 and 100 IU/dL. Not surprisingly, the highest proportional overestimation was seen at low emicizumab concentrations and high FVIII levels. In addition, there was a high variability between aPTT reagents and recombinant FVIII concentrates. However, the residual FVIII after EDTA pretreatment is significantly lower and is therefore unlikely to have a clinically relevant influence on the emicizumab plasma levels measured with mOSA.

Second, we were able to show a high correlation between emicizumab plasma levels measured in citrated plasma and EDTA. The variation between the emicizumab plasma concentration measured in citrated plasma and EDTA on the same platform is comparable with the variation between different platforms with the same anticoagulant. We conclude that the emicizumab plasma concentration can be assessed in EDTA plasma and the results are comparable with the values obtained in citrated plasma. It has been shown that antibodies can be stored in EDTA [11,12], a finding we confirm with our data for emicizumab. Due to the high sample dilution of 1:320 in mOSA, resulting in an EDTA concentration of approximately 25 μmol/L compared with the 250× higher calcium concentration of approximately 6250 μmol/L in the assay, EDTA does not appear to have a negative influence on the test results. Therefore, emicizumab can be quantified via its FVIIIa mimetic function in EDTA.

This study has several limitations. We performed our analysis with a limited number of blood samples, included only 1 reagent on 2 platforms and did not perform measurements under conditions such as hemolysis. Therefore, our results should be confirmed in a larger cohort of patients and using various aPTT reagent on more platforms. We did not validate this approach for FVIII products with modified molecular composition. It is not unlikely that modified single-chain FVIII molecules may show higher stability in EDTA. However, this approach has several advantages. It does not require specialized equipment and trained personnel or sample pretreatment. Further, citrated plasma might not always be available, but EDTA-treated blood samples are usually obtained to measure blood counts. Further research should therefore investigate the stability of emicizumab in EDTA under laboratory conditions. This approach can potentially be transferred to other antibody-based therapies for the treatment of hemophilia.

In conclusion, emicizumab plasma concentration can be measured in EDTA without a relevant influence of residual FVIII. The residual FVIII activity can be measured in citrated plasma using a chromogenic assay with bovine substrates that is not affected by emicizumab. The results of our study provide the basis for an easy approach to measure the emicizumab plasma concentration in the presence of FVIII as well as the residual FVIII activity. This concept can potentially be transferred to other antibody-based therapies for the treatment of bleeding disorders.
