# Supplementary material for: Assessment of emicizumab levels in EDTA plasma
Source: Res Pract Thromb Haemost. 2025 Sep 5;9(6):103175. doi: 10.1016/j.rpth.2025.103175 (PMC12509097; doi:10.1016/j.rpth.2025.103175)

# Supplementary material

Test procedure of the emicizumab assay on COAG360 and CN6000:

Emicizumab assay (Siemens Atellica COAG360)

- 1:8 sample predilution in Owren’s Veronal Buffer
- 40µL of factor VIII deficient plasma
- 40µL of 1:10 dilution of prediluted sample in Owren’s Veronal Buffer
- 2 minutes incubation at 37°C
- 40µL of Actin FS
- 3 minutes incubation at 37°C
- 40µL of 25 mM CaCl_2_
- measurement

Emicizumab assay (Sysmex CN6000)

- 1:8 sample predilution in Owren’s Veronal Buffer
- 1:10 dilution of prediluted sample in Owren’s Veronal Buffer
- 40µL of 1:2 dilution of diluted sample in Owren’s Veronal Buffer
- 40µL of factor VIII deficient plasma
- 40µL of prediluted sample
- 2 minutes incubation at 37°C
- 40µL of Actin FS
- 3 minutes incubation at 37°C
- 40µL of 25 mM CaCl_2_
- measurement

Supplementary table 1: Emcizumab plasma concentration (µg/mL) in citrated plasma and EDTA and relative difference between EDTA compared to citrated plasma measured on COAG 360 and CN-6000.

|  |  | COAG 360 | | | CN-6000 | | |
| --- | --- | --- | --- | --- | --- | --- | --- |
| Plasma sample | Disease | Citrated plasma | EDTA | Relative difference | Citrated plasma | EDTA | Relative difference |
| 1 | HAI | 7.8 | 7.2 | -4.0% | 6.7 | 8.0 | 8.8% |
| 2 | AHA | 18.9 | 16.6 | -6.5% | 17.2 | 15.6 | -4.9% |
| 3 | MHA | 20.8 | 19.5 | -3.2% | 19.9 | 22.0 | 5.0% |
| 4 | HAI | 35.1 | 34.2 | -1.3% | 34.4 | 37.6 | 4.4% |
| 5 | SHA | 38.0 | 36.2 | -2.4% | 47.3 | 46.9 | -0.4% |
| 6 | SHA | 41.2 | 39.2 | -2.5% | 48.2 | 50.7 | 2.5% |
| 7 | HAI | 42.2 | 43.9 | 2.0% | 43.8 | 48.2 | 4.8% |
| 8 | SHA | 42.8 | 43.8 | 1.2% | 41.0 | 45.6 | 5.3% |
| 9 | SHA | 48.3 | 48.6 | 0.3% | 48.7 | 46.4 | -2.4% |
| 10 | SHA | 50.2 | 47.1 | -3.2% | 50.3 | 49.1 | -1.2% |
| 11 | MHA | 52.7 | 54.3 | 1.5% | 58.6 | 55.1 | -3.1% |
| 12 | AHA | 57.6 | 54.4 | -2.9% | 52.3 | 56.4 | 3.8% |
| 13 | SHA | 58.6 | 58.7 | 0.1% | 62.0 | 65.7 | 2.9% |
| 14 | SHA | 60.1 | 54.8 | -4.6% | 77.7 | 76.9 | -0.5% |
| 15 | SHA | 61.2 | 62.1 | 0.7% | 56.4 | 64.0 | 6.3% |
| 16 | SHA | 64.5 | 60.7 | -3.0% | 74.6 | 74.0 | -0.4% |
| 17 | HAI | 67.7 | 67.8 | 0.1% | 70.5 | 66.2 | -3.1% |
| 18 | MHA | 69.3 | 67.9 | -1.0% | 65.6 | 65.6 | 0.0% |
| 19 | SHA | 70.2 | 68.0 | -1.6% | 63.4 | 67.9 | 3.4% |
| 20 | SHA | 84.4 | 83.8 | -0.4% | 77.1 | 83.4 | 3.9% |
| 21 | SHA | 88.9 | 87.7 | -0.7% | 82.0 | 87.1 | 3.0% |
| 22 | SHA | 89.8 | 89.7 | -0.1% | 82.3 | 85.0 | 1.6% |
| 23 | SHA | 101.0 | 101.0 | 0.0% | 91.9 | 94.3 | 1.3% |
| 24 | HAI | 104.0 | 101.0 | -1.5% | 87.9 | 101.3 | 7.1% |
| 25 | SHA | 105.0 | 107.0 | 0.9% | 95.2 | 105.0 | 4.9% |

Abbreviations: HAI: hemophilia A with inhibitor; SHA: severe hemophilia A; MHA: moderate hemophilia A, AHA: acquired hemophilia A.


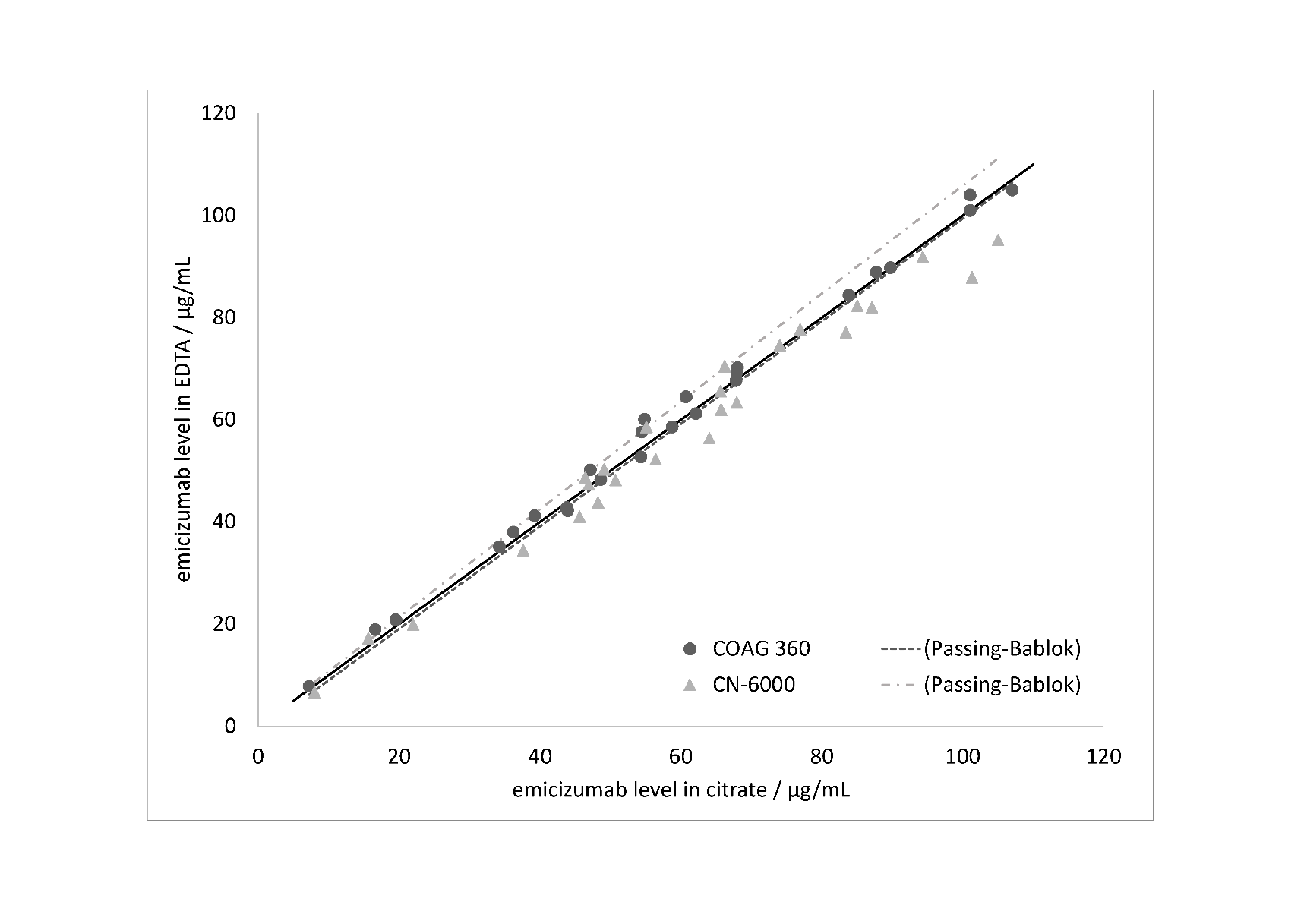
Supplementary figure 1: Passing Bablok plots for the measurement of emcizumab plasma levels in EDTA and citrated plasma on COAG 360 and CN-6000

Supplementary figure 2: Emicizumab plasma concentration measured in citrated plasma and EDTA on COAG 360 and CN 6000 in 25 plasma samples from 16 patients on prophylaxis with emicizumab.


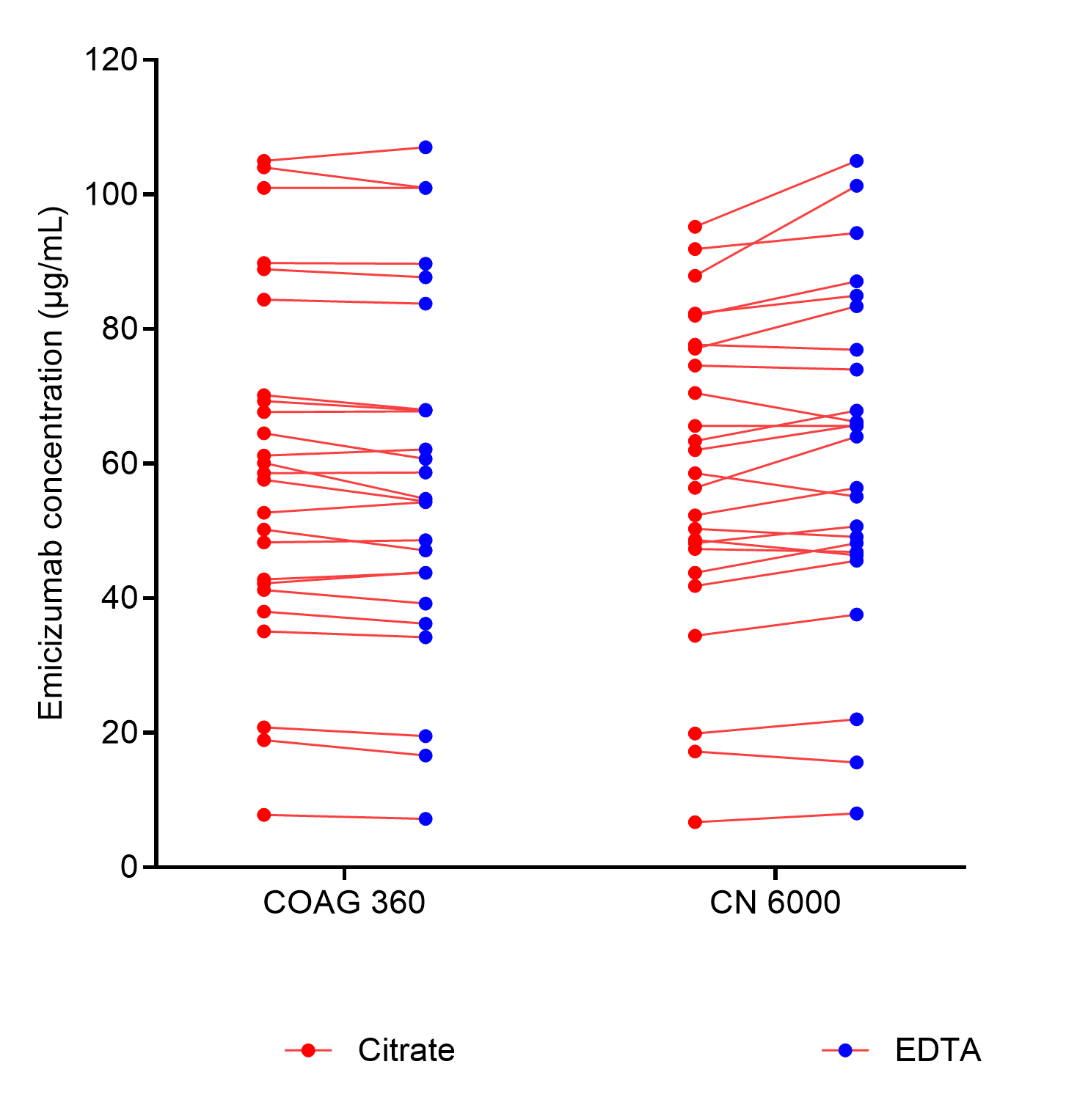

Supplement: Supplementary file 1 [file mmc1.docx]
